# Relationship between Obesity and Cognitive Function in Young Women: The Food, Mood and Mind Study

**DOI:** 10.1155/2017/5923862

**Published:** 2017-10-08

**Authors:** Rebecca L. Cook, Nicholas J. O'Dwyer, Cheyne E. Donges, Helen M. Parker, Hoi Lun Cheng, Katharine S. Steinbeck, Eka P. Cox, Janet L. Franklin, Manohar L. Garg, Kieron B. Rooney, Helen T. O'Connor

**Affiliations:** ^1^Discipline of Exercise and Sport Science, Faculty of Health Sciences, University of Sydney, Lidcombe, NSW, Australia; ^2^School of Human Movement Sciences, Charles Sturt University, Bathurst, NSW, Australia; ^3^Charles Perkins Centre, University of Sydney, Camperdown, NSW, Australia; ^4^Discipline of Paediatrics and Child Health, The Children's Hospital at Westmead, University of Sydney, NSW, Australia; ^5^Metabolism and Obesity Services, Royal Prince Alfred Hospital, Camperdown, NSW, Australia; ^6^Nutraceuticals Research Program, School of Biomedical Sciences & Pharmacy, University of Newcastle, Callaghan, NSW, Australia

## Abstract

Limited research addresses links between obesity and cognitive function in young adults. *Objective*. To investigate the relationship between obesity and cognitive function in young women. *Methods*. This cross-sectional study recruited healthy, young (18–35 y) women of normal (NW: BMI = 18.5–24.9 kg·m^−2^) or obese (OB: BMI ≥ 30.0 kg·m^−2^) weight. Participants completed a validated, computer-based cognitive testing battery evaluating impulsivity, attention, information processing, memory, and executive function. Questionnaires on depression and physical activity and a fasting blood sample for C-reactive protein and the Omega-3 Index were also collected. Cognition data are presented as *z*-scores (mean ± SD), and group comparisons were assessed via ANOVA. Potential confounding from questionnaire and blood variables were evaluated using ANCOVA. *Results*. 299 women (NW: *n* = 157; OB: *n* = 142) aged 25.8 ± 5.1 y were enrolled. Cognition scores were within normal range (±1 *z*-score), but OB had lower attention (NW: 0.31 ± 1.38; OB: −0.25 ± 1.39; ES: 0.41, CI: 0.17–0.64; *p* < 0.001) and higher impulsivity (NW: 0.36 ± 1.14; OB: −0.07 ± 1.07; ES: 0.39, CI: 0.15–0.62; *p*=0.033). Confounder adjustment had minimal impact on results. *Conclusion*. The OB group had normal but significantly lower performance on attention and were more impulsive compared to NW participants. This may indicate early cognitive decline, but longitudinal research confirming these findings is warranted.

## 1. Introduction

Young women are at increased risk of weight gain as they transition into adulthood. Contributing factors include lifestyle changes such as moving away from the family home, cohabitation with peers or partners, increased takeaway food and alcohol consumption, and decreased physical activity [1–3]. For some young women, pregnancy is also a risk factor for weight gain [4]. In Australia, close to one-third of young (18–23 y) women are overweight or obese, and as a group, they are gaining weight faster than older women and young men [3, 5, 6]. By 2025, modeling predicts that one in six Australian women compared to one in 10 men will have severe obesity [7]. Aside from the known metabolic and psychological health risks [8], obesity also affects reproductive health via increased risks for infertility and obstetric complications [9–11]. Growing evidence also indicates a negative impact of maternal obesity on the health of subsequent generations through epigenetic effects on offspring [9, 10, 12, 13]. These risks and the greater rate of weight gain in young Australian women compared to young men indicate a need for targeted research and intervention in this population.

The health consequences of obesity extend to cognitive function, with evidence for reduced memory [14, 15] and executive function [16–19] as well as increased impulsivity [20–22]. Several recent systematic reviews have found that, because of numerous methodological limitations, it is unclear whether the association between obesity and cognitive impairment is independent of obesity-related comorbidities [16–19, 23]. Equivocal findings are likely attributable to poor study design, small sample size, lack of consideration for confounders, and the heterogeneity of psychometric tests used [24].

Proposed mechanisms underpinning reduced cognitive function in obesity include oxidative stress, hypertension, metabolic dysfunction, cardiovascular disease, and systemic inflammation, which have been reported to alter brain structure and volume [24–27]. However, young adults with obesity often have no apparent metabolic and cardiovascular abnormalities although systemic inflammation is usually present which may act directly to impair cognition [28, 29]. Alternatively, reduced physical activity may be important. Physical activity is reported to have beneficial effects on cognitive function, with adequate activity levels shown to be associated with an improvement in cognitive function and attenuation of cognitive decline [30, 31]. In recent years, 57% of Australian adults failed to meet physical activity guidelines [32], and moreover, women are less likely than men to meet recommended levels [33], a trend that begins during adolescence and continues into adulthood [34]. Finally, substantial evidence exists in the literature for an association between blood fatty acids and cognitive impairment, particularly in Alzheimer's disease [35, 36]. An increasing number of studies suggest that the omega-3 polyunsaturated fatty acids docosahexaenoic acid (DHA) and eicosapentaenoic acid (EPA) are protective against cognitive impairment and dementia [37–41]; however, young Australian women have been shown to have inadequate intake of omega-3 fatty acids [42, 43], and low blood omega-3 levels have been commonly observed in young women with depression [44] and during pregnancy [45].

The majority of current research is focused on middle-aged cohorts who have other potential age-related factors influencing cognition. As such, the association between obesity and cognition in younger, healthier adults is equivocal [16–18, 24], particularly with respect to the specific cognitive domains most affected [17, 19]. In line with previous limitations identified in cognition research [17, 23], inconsistencies related to poor study design, small sample size, limited consideration of confounders, and the array of psychometric tests used make it difficult to compare studies, and additionally, there are only a limited number of studies specifically examining young adults [15, 17]. Hence, the current study investigated healthy, young women and hypothesized that young women with obesity would have significantly lower cognitive function, across the memory, executive function, and impulsivity (more impulsive) domains. We further hypothesized that decreased cognitive performance would be ameliorated after adjustment for systemic inflammation. A detrimental impact of obesity on cognition in young adulthood might influence not only the capacity to function optimally at this important age stage but also the risk for earlier cognitive decline [19]. Better evidence of reduced cognition in young adults with obesity would also support advocacy for targeted public health programs aimed at addressing weight gain during this age stage, particularly in women who are gaining weight more rapidly than men [3, 5], and have specific obesity-related reproductive health risks [9, 10, 12, 13].

## 2. Methods

### 2.1. Study Design and Participants

The primary aim of this cross-sectional study, the Food, Mood and Mind study, was to compare cognitive function in young (18–35 y), healthy women of either normal weight (NW) or obese weight (OB). A secondary aim was to investigate the influence of potential confounders including systemic inflammation, omega-3 index (O3I), depression symptoms, and physical activity on cognitive function. In light of the methodological limitations of existing studies highlighted in recent reviews [12–16], Prickett et al. [17] identified five key design considerations for studies of the relationship between obesity and cognition: control of confounding variables (e.g., age, education, and depression); appropriate study design (e.g., relevant participant exclusions and adequate sample size); appropriate control groups; assessment of cognitive domains relevant to the specific research question; and appropriate, valid, and reliable cognitive assessment tools. All of these design features have been incorporated in the current study.

Participants were recruited from both urban/metropolitan (Sydney) and rural/regional (Bathurst) areas in Australia. To ensure adequate representation from regional participants, one-third of the sample was recruited from the rural/regional site. A multipronged approach identified as effective for recruiting young adults to research was employed [46], including flyers, websites, newspaper advertisements, e-newsletters, social media, radio, and letterbox drops. The study was approved by the Human Research Ethics Committees of the local health district (HREC/10/RPAH/455), the University of Sydney (protocol number: 2014/050), and Charles Sturt University (protocol number: X10-0259). Written informed consent was obtained from all volunteers prior to study participation. Participants who completed the study received a gift card ($100 AUD) to cover time and travel costs.

### 2.2. Eligibility Criteria

Volunteers were initially screened for eligibility via telephone using a standardized medical questionnaire, with eligible individuals reporting no medical conditions or medication use on a regular basis (oral contraceptive pill and asthma medications were allowed), and a BMI in the normal weight (18.5–24.9 kg·m^−2^) or obese weight (≥30.0 kg·m^−2^) category according to the World Health Organization guidelines [47]. No volunteers in the overweight category (BMI = 25.0–29.9 kg·m^−2^) were recruited so as to establish a substantial BMI gap for detection of significant cognitive differences. An upper age limit of 35 y was also applied to reduce confounding from age-related cognitive decline [48]. The standardized medical screening questionnaire sought to exclude those with significant medical conditions (e.g., cardiovascular or metabolic diseases including type 2 diabetes) and conditions which may compromise the assessment of cognitive function or the assessment of BMI: (1) neurological or psychiatric conditions and use of medications/substances known to alter mood, reaction time, or cognitive capacity including smoking, alcohol consumption (≥50 g per week), and recreational drug use; (2) vision, hearing, or motor coordination problems and poor English literacy, which may impair the ability to complete the touchscreen cognition testing; and (3) pregnancy, breastfeeding, elite athletes (due to increased muscular development), and regular blood donations (≥3 per year or having donated blood within the previous three months) as iron deficiency/anemia has also been associated with impaired cognitive function [49]. A specialist endocrinologist (Katharine S. Steinbeck) oversaw the recruitment process and provided a medical opinion on participant eligibility prior to their entry to the study.

### 2.3. Data Collection

Participants attended two study visits (at a university laboratory or an obesity clinic within a major teaching hospital) approximately one week apart. The first study visit included anthropometric assessment, followed by assessment of physical activity (International Physical Activity Questionnaire Short Form, IPAQ-SF) [50, 51] and then by assessment of depression (Depression, Anxiety and Stress Scale, DASS) [52] and cognitive function (IntegNeuro^TM^) [53]. All cognitive assessments were conducted after breakfast and prior to 13:00 hrs. Participants were asked to refrain from heavy exercise, alcohol, and caffeine (and other stimulants) on the morning of the cognition test and to consume their usual breakfast. The second visit involved collection of a fasting blood sample. Blood analysis was used to help exclude or adjust for parameters known to influence cognitive function. Fasting blood glucose was analyzed to assist in the exclusion of women with diabetes. Measurement of C-reactive protein (CRP) and omega-3 (n-3) polyunsaturated fatty acid status (via the Omega-3 Index) enabled cognitive function to be adjusted for these potential confounders. See Blood Collection and Biochemical Analysis for further details.

### 2.4. Anthropometry

Anthropometric measurements were taken in light clothing (no shoes). Height was measured to the nearest 0.1 cm in duplicate with a stadiometer (Seca 213; Seca, Hamburg, Germany). Weight was recorded on a digital platform scale accurate to 0.1 kg (PW-200KGL; A&D Weighing, Thebarton, Australia). Waist circumference was measured at the midpoint between the lowest rib and the iliac crest to the nearest 0.1 cm in duplicate (mean reported) with a retractable metal tape (Lufkin W606PM; Cooper Industries, Sparks, USA), according to the International Diabetes Federation guidelines [54].

### 2.5. Cognitive Assessment

Cognitive function was assessed using a computer-based testing platform (IntegNeuro; Brain Resource Company, Woolloomooloo, Australia), using a touchscreen and headset. This platform has well-established validity, reliability, cross-cultural consistency, and norms [55, 56]. The five cognitive domains impulsivity, attention, information processing, memory, and executive function were assessed. The tests for each domain and the specific performance measures for each test are summarized in Table 1 [53]. The measures in each domain were expressed as norm-based *z*-scores, with values between ±1 classified as within normal range, and then averaged to obtain a single *z*-score for each domain. Positive and negative *z*-scores reflect above- and below-average performance, respectively (for impulsivity, positive scores reflect less impulsive behavior, whereas negative scores reflect more impulsive behavior), and are adjusted for age and education using internal regression methods based on Brain Resource Company's extensive normative databank [53]. Participants completed the cognitive assessment using their dominant hand seated in a quiet location.

### 2.6. Depression and Physical Activity Assessment

Symptoms of depression were measured using the DASS [52], administered electronically as part of the IntegNeuro testing platform. Results are expressed as *z*-scores, with positive scores indicating greater depressive symptoms. Physical activity level was assessed via the IPAQ-SF, which estimates duration (in minutes) and frequency (days) of walking, moderate and vigorous activity, and sitting hours per weekday [50, 51]. Physical activity (PA) data are expressed as metabolic equivalent of task (MET) minutes per week.

### 2.7. Blood Collection and Biochemical Analysis

The second study visit involved a morning fasted (12 h) venous blood draw. Blood analyses were undertaken by a NATA accredited laboratory. Inflammation was assessed via CRP, with levels > 5.0 mg/l indicative of elevated inflammation [57, 58]. The O3I was used as a reliable indicator of overall omega-3 (n-3) polyunsaturated fatty acid status, calculated as the sum of the percentage of eicosapentaenoic acid (20:5n-3) and docosahexaenoic acid (22:6n-3) in erythrocyte membranes, as reported elsewhere [59, 60]. Proposed cutoffs for cardiovascular health were used: O3I of <4% is classified as low, 4–8% as safe, and >8% as optimal [60]. Fasting blood glucose was also measured to ensure exclusion of women with diabetes (≥7.0 mmol/l) [61].

### 2.8. Statistical Analysis

Analyses were carried out using Statistica Version 12 (StatSoft Inc., Tulsa, USA). All data were checked for normality and outliers, with cognition scores ±4 standard deviations (SDs) excluded (*n* = 11). Unpaired *t*-tests and chi-square tests were used to compare participant characteristics between the NW and OB groups. The relationship between BMI and cognitive function was initially investigated via a 2 × 5 analysis of variance (ANOVA) model, using the BMI group (NW or OB) as the independent factor and the five cognition *z*-scores (i.e., impulsivity, attention, information processing, memory, and executive function) as repeated measures. This model was rerun as separate analyses of covariance (ANCOVA) models using the covariates DASS score, PA, CRP, and O3I. Finally, the relative contribution of these confounders to the overall BMI-cognition relationship was assessed using a single ANCOVA model which included all the four covariates combined. Tukey's post hoc tests were used in all cases to determine the precise locus of any significant difference observed. Since the cognition domains in the IntegNeuro test battery have been shown via principal component analysis to represent independent factors [62], univariate analyses were further conducted to examine group differences on the cognition *z*-scores. Significance was set at *p* < 0.05, with results reported as mean ± SD unless indicated otherwise and effect size (ES) ± 95% confidence interval (CI).

## 3. Results

### 3.1. Participant Characteristics

**Figure 1:**
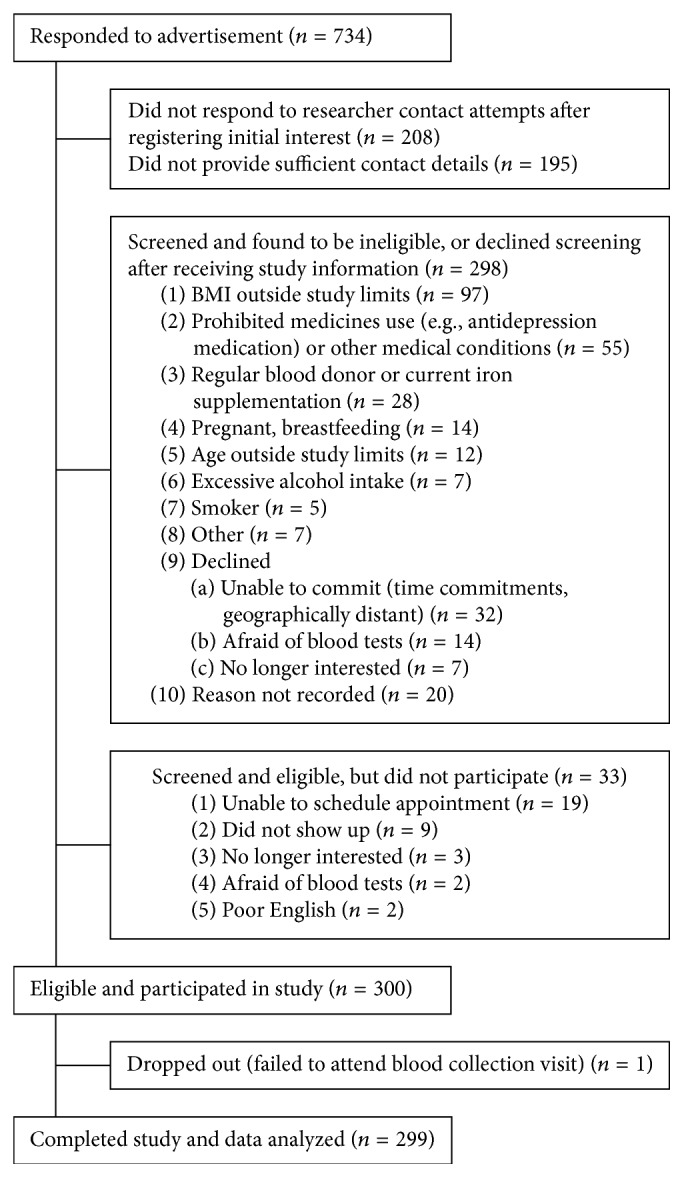
Flow diagram for recruitment, eligibility screening, and participation in the Food, Mood and Mind study.

A total of 300 women were recruited, with 299 completing both study visits (NW: *n* = 157; OB: *n* = 142); one participant failed to attend the second study visit (Figure 1()). Major reasons for exclusion included BMI outside the study limits (32%), depression or anxiety medications or significant medical condition (15%), and time constraints or geographical location (11%).

Participant characteristics are summarized in Table 2. Average age of the participants was 25.8 ± 5.1 y although the OB group was slightly but significantly older by ∼2 y (*p* < 0.001). A majority of participants (57%) held tertiary qualifications, with the mean years of education being 16.2 ± 2.2 y. Average time spent in education (∼0.6 years less; *p*=0.022) and the proportion of tertiary educated women (11% less; *p*=0.041) were both lower in the OB group. The majority of participants in the OB group had class I obesity (55%), with smaller proportions having class II (29%) and III (16%) obesity. The majority of the OB group (97%) had a waist circumference above the current recommendation (≥80 cm) to reduce risk of metabolic disorders and other comorbidities [54]. A substantial proportion (85%) had a waist circumference classified as very high risk of chronic disease (≥88 cm) [54]. The high waist circumference of the participants in the OB group confirms that their BMI was due to excess adiposity rather than increased muscular development.

### 3.2. Inflammation, Physical Activity, and Omega-3 Index

The OB group showed significantly higher CRP levels (NW: 1.4 ± 2.1; OB: 5.5 ± 5.0 mg/l; *p* < 0.001) and significantly lower PA levels (NW: 3076 ± 2302; OB: 2080 ± 1816 MET-min/wk; *p* < 0.0001) than the NW group (Table 2). Most participants (64%) met the American College of Sports Medicine guidelines for moderate to vigorous PA (500 MET-min/wk) [63], although fewer participants in the OB group met these guidelines (NW: 74%; OB: 54%; *p* < 0.001). Mean O3I was lower in the OB group (*p* < 0.001), with a greater proportion of participants showing low O3I status (*p*=0.001) and a smaller proportion having optimal O3I (*p*=0.029).

### 3.3. Obesity and Cognition

**Figure 2:**
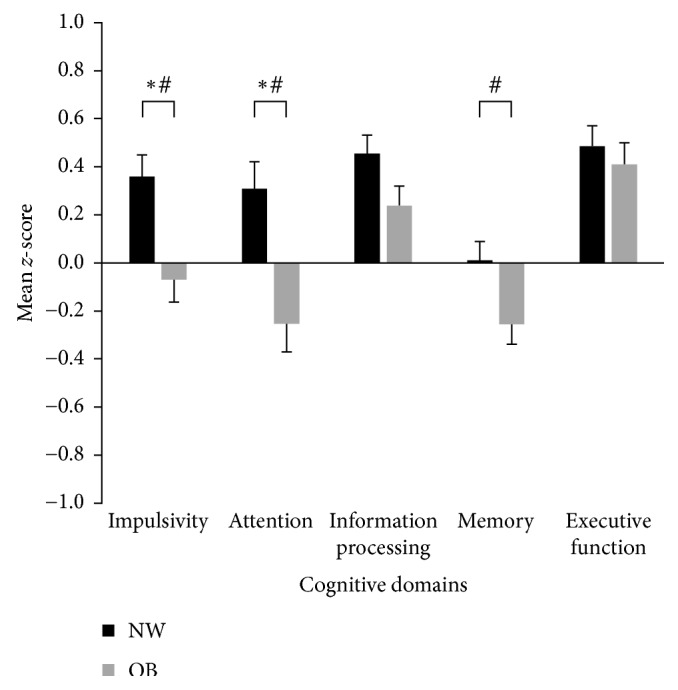
Comparison of cognition *z*-scores assessed across five domains between young women categorized into NW and OB groups. Domain scores adjusted for age and education, with data presented as mean ± standard error. Normal range is between ±1. ^∗^*p* < 0.05 between NW and OB in post hoc analyses; ^#^*p* < 0.05 between NW and OB in univariate analysis. Lower scores on the impulsivity domain indicate increased impulsive responses. BMI, body mass index; NW, normal weight (BMI = 18.5–24.9 kg/m^2^); OB, obese weight (BMI ≥ 30.0 kg/m^2^).

In both the NW and OB groups, mean *z*-scores for all the five cognitive domains (impulsivity, attention, information processing, memory, and executive function) were within the normal range (i.e., ±1 *z*-score) (Figure 2()). The group mean values (±95% confidence intervals) are presented in Table 3. Analysis of cognitive performance showed a significant overall between-group difference (*p* < 0.001) and a significant interaction between groups and domains (*p*=0.028). Post hoc analyses identified lower scores in the OB group for impulsivity (NW: 0.36 ± 1.14; OB: −0.07 ± 1.07; *p*=0.033; ES: 0.39, CI: 0.15–0.62; lower scores indicate greater impulsivity) and attention (NW: 0.31 ± 1.38; OB: −0.25 ± 1.39; ES: 0.41, CI: 0.17–0.64; *p* < 0.001). Univariate analyses showed significantly poorer performance for impulsivity (*p*=0.001), attention (*p*=0.0007), and memory (*p*=0.022) in OB relative to NW, as well as a nonsignificant trend for lower information processing (*p*=0.055).

### 3.4. Influence of Known Confounders on Cognition

Adjustment for DASS score and PA in separate ANCOVA models did not alter the significant group effect (DASS: *p* < 0.001; PA: *p* < 0.001) or interaction effect (DASS: *p* < 0.01; PA: *p* < 0.019) observed between BMI and cognitive domains. Adjustment for CRP and O3I weakened the interaction effect (CRP: *p*=0.060; O3I: *p*=0.067), but a significant overall difference between BMI groups remained (CRP: *p*=0.003; O3I: *p* < 0.001). Adjusted post hoc analyses yielded results similar to the unadjusted ANOVA, with significantly greater impulsivity (i.e., a poorer/lower score in the impulsivity domain) in the OB group after adjustment for DASS score (*p*=0.033), PA (*p*=0.034), and CRP (*p*=0.043), but not for O3I (*p*=0.12), and significantly lower attention following adjustment for the four covariates (all *p* < 0.001). After adjusting for each of the four covariates, univariate analyses showed that performance remained significantly lower in the OB group than in the NW group on impulsivity (DASS: *p*=0.002; PA: *p*=0.001; CRP: *p*=0.004; O3I: *p*=0.004) and attention (DASS: *p* < 0.001; PA: *p* < 0.001; CRP: *p*=0.005; O3I: *p*=0.002), with memory remaining significant for all except CRP (DASS: *p*=0.013; PA: *p*=0.007; CRP: *p*=0.19; O3I: *p*=0.027). These effects remained largely unchanged when a final ANCOVA model combining the four covariates was carried out. After adjustment for all potential confounders, the reduction of cognitive performance in the OB group in the impulsivity and attention domains was 0.38 and 0.59 SD, respectively.

## 4. Discussion

This cross-sectional study found that while cognition was within normal range, healthy young women with obesity achieved significantly lower performance in the attention and impulsivity domains compared to their NW peers, with evidence of lower performance also in the memory domain. Adjustment for known confounders had minimal impact on these findings although there was some attenuation of BMI-related differences for impulsivity and memory. Diminished cognitive performance in young women with obesity may indicate the beginning of a persistent and early cognitive decline. However, longitudinal research is required to confirm our findings, and studies including a more socioeconomically diverse sample are warranted. Despite this, our rigorous exclusion criteria suggest that a true cognitive deficit exists in OB individuals, especially as our sample included highly educated women who would be expected to be at their peak age for cognitive function.

Lower performance in the OB group for the attention, impulsivity, and memory domains differs from reports in the literature. Contrary to existing studies that link obesity with poorer executive function [16–19, 24], our scores obtained for this domain were found to be the most similar between the OB and NW groups (Figure 2()). This discrepancy may be attributed to the way that cognitive tests are classified. Attention and impulsivity, which were tested separately and where we found clear BMI group differences, are sometimes categorized under executive function [16, 17, 64]. The trail making test, for example, which is classified under information processing in the IntegNeuro platform used here, has usually been classified under executive function, with mostly nonsignificant findings reported for BMI groups in recent reviews [16–19, 24]. Executive function was assessed in the current study using the maze test, and the only other comparable study using this same test employed it in a cross-sectional study examining cognition across the adult lifespan (*n* = 408; 20–82 y) [64]. These researchers found poorer performance in both younger and older overweight/obese adults (BMI > 25) compared to NW adults [64], but it should be noted that the age range of their “younger” group was 20–50 years and hence, unlike the present study, the influence of age-related comorbidities could not be ruled out.

Attention was consistently and significantly lower in OB participants. This finding is in agreement with a study by Cserjési et al. reporting lower attention in middle-aged women with obesity compared to nonobese controls [65]. Both our study and that of Cserjési et al. utilized the continuous performance test, which measures sustained attention [53, 65]. Gunstad et al. reported no significant differences between NW and overweight/obese adults on four tests classified under the attention domain, including switching of attention (similar to the trail making test [53]), choice reaction time, digit span forward, and span of visual memory [64]. Switching of attention and choice reaction time tests are classified under the information processing domain in the IntegNeuro platform. Hence, our finding of no significant differences between BMI groups on these tests is in agreement with the results of Gunstad et al. [64].

There is increasing evidence of an association between obesity and attention disorders such as attention-deficit hyperactivity disorder (ADHD) [66]. In fact, a reward deficiency syndrome has been identified in obesity and ADHD, which results in insufficient dopamine-mediated “natural” rewards, leading to the seeking of “unnatural rewards” such as risk taking, gambling, and uncontrolled eating [67, 68]. Dysfunctional attention and impulsivity in ADHD may lead to obesity via abnormal eating behaviors [66]. Obesity was also found to be significantly associated with impulsivity in the current study, using the go/no-go task to assess cognitive inhibition (this test assesses the capacity to suppress well-learned, automatic responses). Other studies which have employed the Iowa Gambling Task or delay discounting tasks provide further evidence of increased impulsivity in individuals with obesity [22, 69–71].

Literature linking impulsivity and obesity is consistent with studies reporting that individuals with obesity may have difficulty inhibiting automatic or dominant behavior (response inhibition) and delaying gratification [69, 71, 72]. They may have a higher sensitivity to reward compared to normal weight controls [65, 69–72]. Studies have also reported that high impulsivity is related to diminished weight loss during treatment and may predict attrition in those seeking treatment [72, 73]. Addressing deficits in attention and impulsivity may therefore be relevant for weight management interventions [73]. Moreover, reduced inhibition may underlie overeating in obesity [72, 73], and possible shared mechanisms/pathways between decreased attention and impulsivity (as discussed previously) may also explain the lower scores observed in the young women with obesity in this study.

Memory was also identified to be lower in the OB group compared to the NW group, although the association was less strong than for the attention and impulsivity domains (Figure 2()). Post hoc tests were not significant, but the univariate tests for memory remained significant after adjustment for three of the four confounders. The memory tests used in our study were memory recognition (immediate and delayed) and digit span (forward and reverse). Using the memory recognition tests, detrimental effects of obesity on memory were reported previously by Gunstad et al. in their cross-sectional study across the adult lifespan (*n* = 486, 21–82 y) [14]; however, when using the digit span forward test, there were no significant effects in the same population [64]. Leptin and ghrelin dysregulation has been implicated in both memory and obesity, which may account for observed differences in NW versus OB individuals [14, 74–76]. Additionally, while requiring elucidation in humans, elevated BMI has been associated with increased concentrations of brain metabolites such as myoinositol/creatine (MI/Cr) [77]. Such metabolites accumulate in grey matter regions associated with memory and are suggested to indirectly influence memory performance [77]. Overall, the evidence in the literature regarding the effects of obesity on memory function is equivocal. Further research in this area is particularly important as obesity has been linked to an increased risk of dementia [78, 79].

### 4.1. Limitations and Conclusions

Overall, cognitive performance in OB participants was within normal range and was minimally influenced by physical activity, depression symptoms, inflammation, or omega-3 status. Adjusting for these potential confounders did not alter the significant BMI group differences for attention or impulsivity and only partially attenuated OB-related deficits for memory. Such findings indicate that obesity per se may influence cognition independently of these confounders [29, 80]. It is also important to consider that the relationship between obesity and cognition may be in the reverse direction such that specific cognitive attributes increase the risk for obesity [72, 73, 81]. This has certainly been previously proposed and associated in particular with impulsivity and attention as discussed earlier. More research is needed in this area and specifically how different cognitive limitations challenge effective weight management. As the cognitive function was within the normal range, it is difficult to speculate on the functional implications for everyday living or on the clinical significance of these cognitive differences.

This study was designed to examine cognition in young women with obesity and compare their performance with women within the normal weight range. We did not measure women in the overweight range, so we are unable to speculate on the cognitive performance of this “middle group” and this is an important limitation of the study. However, as one of the first studies investigating the association between uncomplicated obesity and cognition in young women, the finding of significant differences in cognitive function supports the need for additional research across the BMI spectrum. An important question for future research is whether a negative relationship (which could be linear or nonlinear) between cognitive performance and BMI applies across the BMI spectrum or, instead, whether there is a BMI threshold above which cognitive performance deteriorates. Future studies may also be able to incorporate brain imaging techniques to elucidate this further and determine if pathological changes are evident above a BMI threshold. Furthermore, given the relationships that have been reported between individual omega-6 as well as omega-3 fatty acids and cognitive decline in older adults and animal models [35, 36, 82, 83], in-depth lipid analysis will benefit future studies in this area. A further limitation of our study is that our focus on young women means that our results are not generalizable to young men. Our decision to focus on young women with obesity was primarily due to the greater rate of weight gain in young women when compared to young men and the prediction that more Australian women than men will be severely obese by 2025 [7].

This study is one of the first to exclude and/or adjust for a range of confounding variables when examining the influence of obesity on cognitive function in young women. The cohort was healthy, well educated, and free of known comorbidities. Exclusion or adjustment was also undertaken for potential confounders. Although the cross-sectional design of the study precludes causal relationships from being determined, our finding of reduced cognitive performance in the attention and impulsivity domains for young women with obesity, and weaker evidence for lower memory function when compared to normal weight women, deserves further examination via longitudinal research. Large epidemiological studies across the BMI spectrum and clinical trials examining the effect of acute and longer-term weight loss on the cognitive function of young women (or young adults) with obesity are warranted.

## Figures and Tables

**Table 1:** Summary of cognitive domains, tests, and measures.

Cognitive domain	Tests	Measures
Impulsivity	Go/no-go	Variability of reaction time (Go)
	Go/no-go	Total errors
Attention	Continuous performance task	Reaction time
	Continuous performance task	False alarm errors
	Continuous performance task	False miss errors
Information processing	Switching of attention	Completion time (digits and letters)
	Switching of attention	Errors (digits and letters)
	Choice reaction time	Reaction time
Memory	Memory recall and recognition	Total immediate recall
	Memory recall and recognition	Long-delay (distractor) recall
	Digit span forwards	Number recalled correctly
	Digit span backwards	Number recalled correctly
Executive function	Maze test	Completion time
	Maze test	Overrun errors

The switching of attention task was the trail making test, part B.

**Table 2:** A summary of participant characteristics.

	Total group (*n* = 299)	NW (*n* = 157)	OB (*n* = 142)	*p* value^∗^
Age (y)	25.8 ± 5.1	24.9 ± 4.6	26.9 ± 5.4	<0.001
Education (y)	16.2 ± 2.2	16.5 ± 2.2	15.9 ± 2.2	0.022
Highest qualification (*n*, %)				
Secondary school	83 (28%)	44 (28%)	39 (27%)	0.028
Certificate/diploma	46 (15%)	15 (10%)	31 (22%)	—
Tertiary degree (bachelor/diploma)	120 (40%)	69 (44%)	51 (36%)	—
Higher degree	49 (17%)	28 (18%)	21 (15%)	—
Weight (kg)	78.1 ± 23.5	59.7 ± 7.0	98.5 ± 17.9	<0.001
Height (cm)	165.4 ± 6.9	165.5 ± 7.3	165.3 ± 6.6	0.805
BMI (kg/m^2^)	28.6 ± 8.6	21.8 ± 1.7	36.1 ± 6.8	<0.001
Obese class I	79 (26%)	N/A	79 (56%)	—
Obese class II	41 (14%)	N/A	41 (29%)	—
Obese class III	22 (7%)	N/A	22 (15%)	—
Waist circumference (cm)	84.5 ± 18.7	69.7 ± 4.2	101.2 ± 14.1	<0.001
Below 80 cm (*n*, %)	156 (52%)	154 (98%)	2 (1%)	<0.001
80–88 cm inclusive (*n*, %)	20 (7%)	3 (2%)	17 (12%)	—
Above 88 cm (*n*, %)	120 (40%)	0 (0%)	120 (85%)	—
Physical activity (MET-min/wk)				
Total MET-min/wk	2603 ± 2141	3076 ± 2302	2080 ± 1815	<0.001
Low MET-min/wk	1137 ± 1190	1246 ± 1234	1017 ± 1131	0.096
Moderate MET-min/wk	499 ± 755	563 ± 809	428 ± 685	0.124
High MET-min/wk	967 ± 1263	1267 ± 1435	636 ± 939	<0.001
CRP (*normal range < 5 mg/l*)	3.4 ± 4.3	1.4 ± 2.1	5.5 ± 5.0	<0.001
CRP < 5 mg/l (*n*, %)	233 (78%)	149 (96%)	84 (60%)	<0.001
CRP ≥ 5 mg/l (*n*, %)	63 (21%)	6 (4%)	57 (40%)	—
Omega-3 index (%)	6.3 ± 1.7	6.8 ± 1.7	5.8 ± 1.6	<0.001
Low: <4% (*n*, %)	13 (4%)	1 (1%)	12 (8%)	0.002
Safe: 4–8% (*n*, %)	239 (83%)	125 (83%)	114 (85%)	—
Optimal: >8% (*n*, %)	36 (12%)	25 (16%)	11 (8%)	—
Depression, Anxiety and Stress Scale (*z*-score)	−0.12 ± 0.88	−0.33 ± 0.71	0.11 ± 0.98	<0.0001

^∗^ *p* value for NW versus OB. Missing data: waist circumference (OB: *n* = 3); O3I (NW: *n* = 6; OB: *n* = 5). Obese class I = 30.0–34.9 kg/m^2^; obese class II = 35.0–39.9 kg/m^2^; obese class III ≥ 40.0 kg/m^2^. BMI, body mass index; N/A, not applicable; PA, physical activity; MET, metabolic equivalent of task; min, minute; wk, week; CRP, C-reactive protein.

**Table 3:** Mean *z*-scores (±95% confidence intervals) for normal weight and obese groups.

Cognition domain	Normal weight		Obese		*p* value^∗^
	Mean	±95% CI	Mean	±95% CI	
Impulsivity	0.36	0.18 to 0.54	−0.07	−0.25 to 0.11	0.001
Attention	0.31	0.09 to 0.53	−0.25	−0.49 to −0.02	0.001
Information processing	0.45	0.31 to 0.60	0.24	0.07 to 0.41	0.055
Memory	0.01	−0.14 to 0.16	−0.25	−0.43 to −0.08	0.022
Executive function	0.49	0.33 to 0.64	0.41	0.22 to 0.60	0.540

^∗^Univariate tests.
